# Ultrasound Findings of Breast Cancer in Dialysis Patients Require Careful Evaluation: A Case Report

**DOI:** 10.7759/cureus.98937

**Published:** 2025-12-10

**Authors:** Mami Yoshida, Shoji Oura

**Affiliations:** 1 Department of Surgery, Kishiwada Tokushukai Hospital, Kishiwada, JPN

**Keywords:** atypical breast image, breast cancer, breast edema, dialysis, peritumoral high echoes

## Abstract

Literature on the effects of renal failure on breast imaging in breast cancer patients on dialysis is scarce. A 73-year-old woman on dialysis noticed a mass in her left breast. In the left breast, which consisted entirely of fat tissue, mammography revealed two spiculated masses. Ultrasound also showed two irregular masses with internal low echoes, slightly attenuated posterior echoes, obscured margins, high depth-to-width ratios, no halos, and widespread peritumoral high echoes. In addition to axillary lymphadenopathy, MRI of the masses showed low signals on T1-weighted images and weakly high signals on fat-suppressed T2-weighted images. Core needle biopsy of the mass pathologically revealed atypical cells growing in trabecular and tubular patterns, with interstitial connective tissue proliferation and fat invasion, leading to a diagnosis of invasive ductal carcinoma. Due to impaired renal function, the patient underwent primary surgery, including a partial mastectomy and axillary lymph node dissection. Postoperative pathological examination showed scirrhous-type invasive ductal carcinomas infiltrating the surrounding fat tissue, nine metastatic lymph nodes, and abundant fat tissue with very scant mammary gland around the tumors. The patient had higher-than-usual postoperative drainage volumes, which varied from day to day, presumably due to the effects of dialysis. Despite a large 100 mL drainage volume on the fifth postoperative day, the patient was discharged on day six per her preference. Diagnostic physicians should note that breast cancer patients on dialysis can present with atypical breast imaging compared with patients without renal failure and should be treated with optimal surgical procedures based on thorough image evaluation.

## Introduction

In many developed countries, breast cancer is the most common malignant tumor in women [1] and is primarily diagnosed using mammography. When the breasts consist entirely of fat tissue or scattered fibroglandular tissue, mammography can accurately depict masses, often enabling the detection of very small breast cancers during screening. Conversely, mammography may sometimes fail to show even large masses when they are present in breasts with either heterogeneous fibroglandular tissue or extremely dense fibroglandular tissue.

Ultrasound is another common diagnostic modality for breast disorders. It can detect small breast masses even in patients with breasts composed of either heterogeneous or extremely dense fibroglandular tissue and is a crucial tool in diagnosing breast diseases. In addition, ultrasound provides valuable information about tumor biology through the evaluation of internal echoes, posterior echoes, margin status, and halos [2-4].

Patients on dialysis can also develop breast cancer and may present with potentially different imaging findings depending on whether tests are performed before or after dialysis, due to dialysis-related weight changes [5]. However, no studies have evaluated the effects of dialysis on breast cancer imaging to date.

We herein report a breast cancer patient on dialysis who presented with characteristic ultrasound images.

## Case presentation

**Figure 1:**
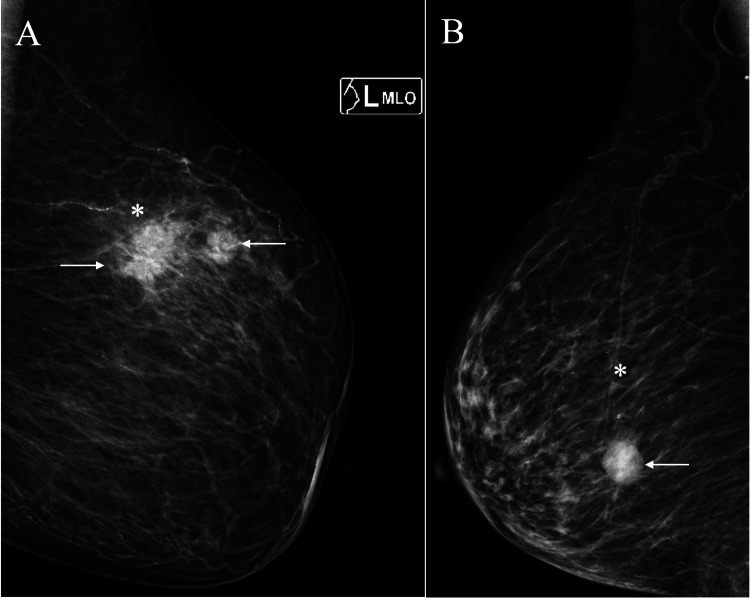
Mammography findings (A) Medio-lateral-oblique view mammography showed two irregular masses (arrows) in the slightly brighter background fat (asterisk). (B) Mammography of another breast cancer patient showed a circumscribed oval mass (arrow) in the darker fat (asterisk) despite a similar background breast composition to the present case.

A 73-year-old woman with renal failure had undergone hemodialysis three times a week for eight years. She noticed a mass in her left breast and visited our hospital. In the left breast, which consisted entirely of fat tissue, mammography revealed two spiculated masses (Figure 1A, 1B).

**Figure 2:**
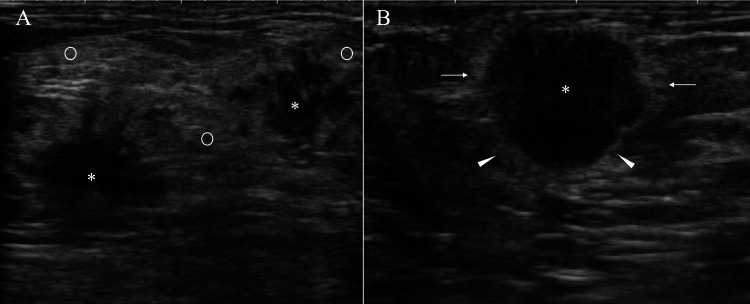
Ultrasound findings (A) Ultrasound showed two irregular masses (asterisks) with internal low echoes, indistinct margins, and widespread high echoes (open circles) around the masses. (B) Ultrasound of another breast cancer patient (the same case as Figure 1B) showed a low-echoic mass with halos not only at the anterior borders of the mass but also at the lateral (arrows) and posterior (arrowheads) borders.

Ultrasound also revealed swollen axillary lymph nodes and two irregular masses with internal low echoes, slightly attenuated posterior echoes, obscured margins, high depth-to-width ratios, no halos, and widespread peritumoral high echoes (Figure 2A, 2B).

**Figure 3:**
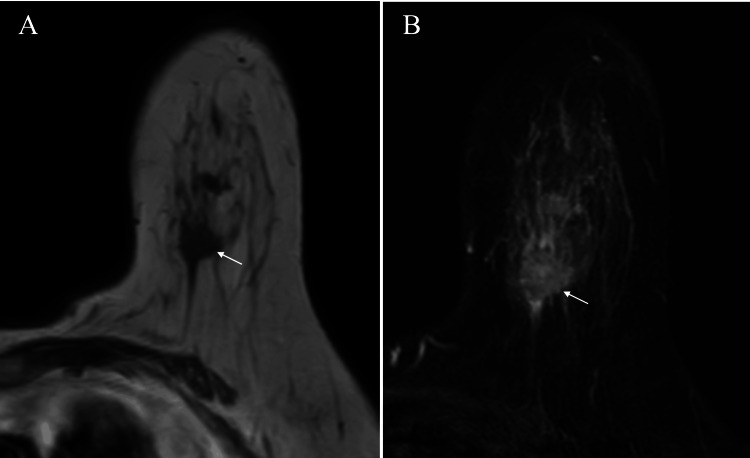
MRI findings MRI of the tumors showed low signals on T1-weighted images (A, arrow) and weakly high signals on fat-suppressed T2-weighted images (B, arrow), highly suggestive of fibrous components within the masses.

In addition to axillary lymphadenopathy, MRI of the masses revealed low signals on T1-weighted images and weakly high signals on fat-suppressed T2-weighted images (Figure 3A, 3B).

We therefore performed a core needle biopsy of the mass under the tentative diagnosis of breast cancer with abundant fibrous components both at the mass margins and within the masses. Pathological examination of the biopsy specimen revealed atypical cells growing in trabecular and tubular patterns, with interstitial connective tissue proliferation and fat invasion, leading to a diagnosis of invasive ductal carcinoma. Immunostaining demonstrated positivity for estrogen and progesterone receptors (ER and PgR), human epidermal growth factor receptor type 2 (HER2), and a Ki-67 labeling index of 30%.

**Figure 4:**
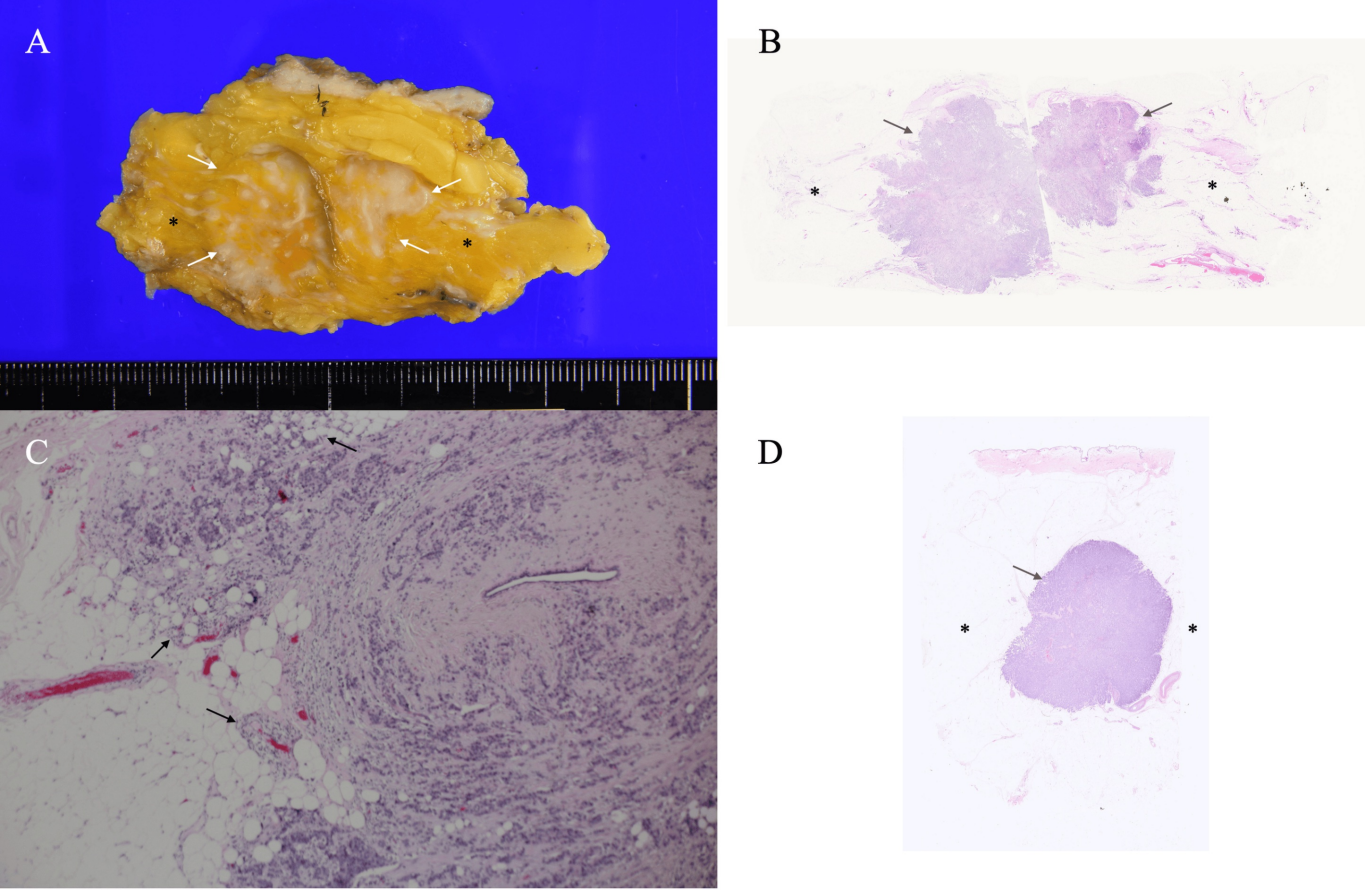
Pathological findings (A) Cut surfaces of the resected specimen showed tumors with indistinct borders (arrows) and very sparse mammary glands around the target lesions (asterisks). (B) Low-magnification view showed two distinct masses (arrows) with only minute atrophic mammary gland remnants around them (asterisks). (C) High-magnification view (H&E ×40) showed cancer cell infiltration into the fat tissue (arrows). (D) A low-magnification view of another case (the same case as Figure 1B and Figure 2B) showed very little mammary gland (asterisks) around the breast cancer (arrows).

Due to impaired renal function, the patient’s poor general condition, and her strong preference, the breast cancer was managed not with neoadjuvant chemotherapy but with primary surgery, including partial mastectomy and axillary lymph node dissection. Postoperative pathological examination revealed scirrhous-type invasive ductal carcinomas infiltrating the surrounding fat tissue, nine metastatic lymph nodes, and abundant fat tissue with very scant mammary gland around the tumors (Figure 4A-4D).

**Figure 5:**
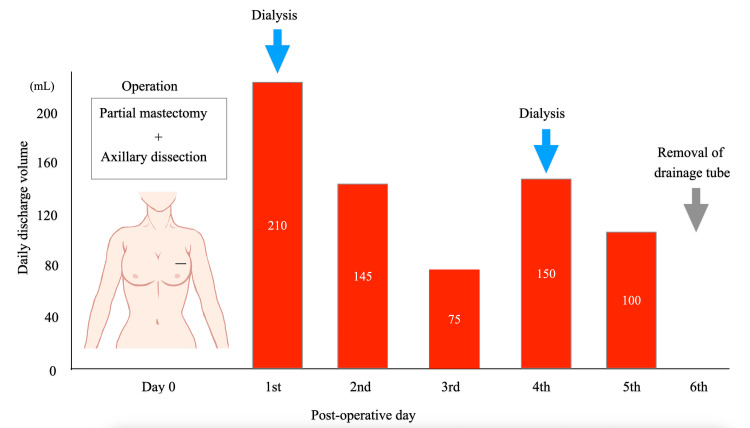
Postoperative daily drainage volumes The patient had a daily drainage volume of 145 mL on the second postoperative day, which decreased markedly to 75 mL on the third day, increased again to 150 mL on the fourth day, and remained high at 100 mL on the fifth day; she was discharged on the sixth day.

Immunostaining of the resected tumors showed similar findings for ER, PgR, and HER2 compared with the core needle biopsy specimen, while the Ki-67 labeling index differed, at 15%. The patient had higher-than-usual postoperative drainage volumes, which varied from day to day, presumably due to the effects of dialysis. Despite a large drainage volume of 100 mL on the fifth postoperative day, the patient was discharged on day six according to her preference (Figure 5).

Shortly after discharge, the patient was unfortunately admitted to another hospital due to dehydration, presumably related to renal failure. She will provide an informed decision regarding whether to receive adjuvant systemic therapy after full recovery.

## Discussion

Medical advances have markedly prolonged the survival of dialysis patients [6]. Attending physicians, therefore, need to be more aware than ever of the occurrence of breast cancer in this population. However, in the treatment of advanced breast cancer, it is often difficult even for oncologists to determine how to apply systemic therapy to patients on dialysis. Early detection is therefore imperative to enable appropriate treatment, given the challenges of systemic therapy in dialysis patients.

In the initial diagnostic step, we interpreted the widespread peritumoral high echoes on ultrasound as reflecting the presence of the mammary gland. Mammography, however, clearly showed that the breast consisted entirely of fat tissue, which differed significantly from our initial evaluation based on ultrasound. In the breast, the extralobular stroma includes surrounding stroma and edematous stroma [7]. It is well known that the latter produces high echoes in normal mammary gland tissue and decreases in volume with aging. Given the patient’s age of 73 years, we conclude that the high echoes around the masses were not due to edematous stroma.

Although pathological findings made it difficult to determine the presence of breast edema directly, it was clear that the widespread peritumoral high echoes did not correlate with the mammary gland, as pathology revealed very sparse mammary tissue around the breast cancers. Additionally, the fat density on mammograms in this patient was slightly brighter than that observed in non-dialysis patients (Figure 1B).

The patient underwent postoperative tube drainage following axillary dissection [8]. Normally, daily drainage volumes after breast cancer surgery gradually decrease over time unless there are drainage tube complications. In this case, the patient had not only larger-than-usual drainage volumes but also fluctuations in daily volumes. It is therefore reasonable to consider that renal failure contributed to breast edema, which in turn affected postoperative drainage volumes through dialysis-related effects.

Breast cancer cell infiltration into fat tissue is known to cause ultrasound wave backscattering due to the extremely low acoustic impedance of fat tissue [9-13], generating band-like high echoes called halos (Figure 2B). The presence and extent of halos can sometimes influence surgical planning in breast cancer surgery. In this case, however, halos could not be identified, presumably due to the breast edema-driven widespread high echoes, despite pathologically proven cancer cell infiltration into adjacent fat tissue.

To date, no studies have reported the effects of dialysis on imaging in breast cancer patients. Comparison of pathological, mammographic, and ultrasound findings in this case strongly suggests that diagnostic physicians should consider breast edema-driven high echoes when evaluating ultrasound images in breast cancer patients on dialysis.

## Conclusions

Dialysis patients can develop renal failure-related edematous changes in the breasts, leading to atypical breast imaging that differs from findings in breast cancer patients without renal failure. Breast edema can generate high echoes even in normal fat tissue and obscure halos in ultrasound evaluation of breast cancer. Diagnostic physicians should therefore recognize that breast cancer patients on dialysis may present with atypical ultrasound findings and plan feasible surgical procedures based on careful ultrasound image assessment.
